# A case of successful conversion surgery for gastric cancer with direct invasion to pancreatic head

**DOI:** 10.1093/jscr/rjab186

**Published:** 2021-05-18

**Authors:** Manabu Sato, Koujin Endo, Akihiko Harada, Masahiro Shijo

**Affiliations:** Department of Surgery, Shiogama City Hospital, Shiogama, Japan; Department of Surgery, JCHO Sendai South Hospital, Sendai, Japan; Department of Surgery, JCHO Sendai South Hospital, Sendai, Japan; Department of Surgery, JCHO Sendai South Hospital, Sendai, Japan; Department of Surgery, JCHO Sendai South Hospital, Sendai, Japan

**Keywords:** gastric cancer, HER2 positive, conversion surgery

## Abstract

We experienced the case of successful conversion to distal gastrectomy for gastric cancer with direct invasion to the pancreatic head after the pre-operative chemotherapy. Esophagogastroduodenoscopy in a 66-year-old man revealed a tumor at the gastric antrum. Abdominal computed tomography (CT) showed that the tumor of the antrum was in contact with the pancreatic head. A biopsy of the tumor confirmed an adenocarcinoma and an overexpression of HER2 (3+). Staging laparoscopy showed the direct invasion of the gastric tumor to the pancreatic head. The patient received S-1, oxaliplatin and trastuzumab. After the pre-operative chemotherapy, CT showed a significantly shrinking tumor detached from the pancreatic head. Subsequently, distal gastrectomy was performed. Intra-operative exploration showed that the gastric tumor separated from the pancreatic head. The accumulation of trials for pre-operative chemotherapy for local advanced gastric cancer is expected.

## INTRODUCTION

In gastric cancer with direct invasion to the pancreatic head, pancreaticoduodenectomy (PD) is necessary to achieve complete margin-negative resection [1]. However, PD has a higher rate of morbidity than gastrectomy alone [1, 2]. The advanced tumor is expected to shrink and become detached from the pancreas with pre-operative treatment.

In the present case, we successfully performed distal gastrectomy to avoid PD after pre-operative chemotherapy for gastric cancer with the overexpression of epidermal growth factor receptor 2 (HER2) directly invading the pancreatic head, and a pathological analysis of the tumor showed dramatic shrinkage.

## CASE REPORT

A 66-year-old man underwent esophagogastroduodenoscopy (EGD) as a routine examination. This revealed an irregularly shaped ulcerated tumor at the greater curvature of the gastric antrum. Blood tests showed a normal peripheral blood count, hepatobiliary function, pancreatic enzyme, renal function, albumin and carbohydrate 19–9 (CA19–9) level. However, the carcinoembryonic antigen (CEA) level was elevated to 64.3 ng/ml (normal range; <5.0 ng/ml).

**Figure 1:**
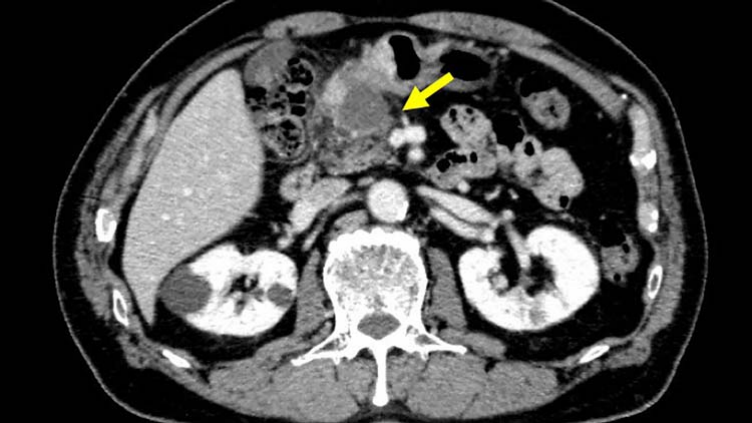
Abdominal CT showed the tumor at the gastric antrum visualized as a thickened wall, suggestive of direct invasion to the pancreatic head (arrow).

**Figure 2:**
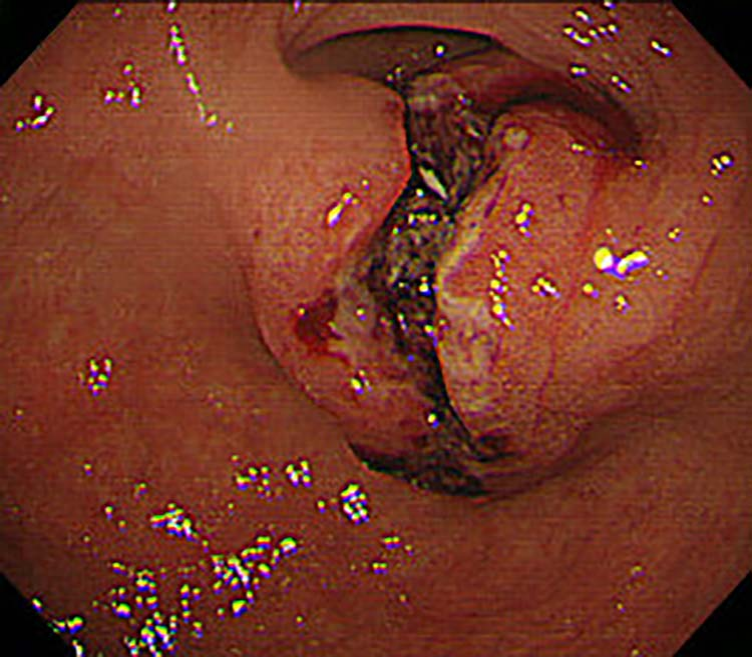
EGD revealed a large ulcerated lesion at the greater curvature of the antrum.

**Figure 3:**
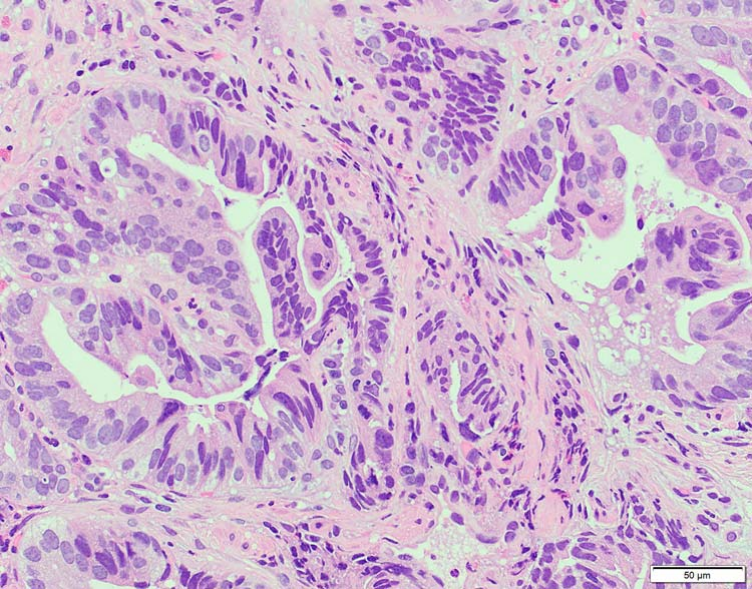
Hematoxylin and eosin staining of the biopsy specimen of the tumor confirmed a well-differentiated adenocarcinoma (tub1, tub2).

**Figure 4:**
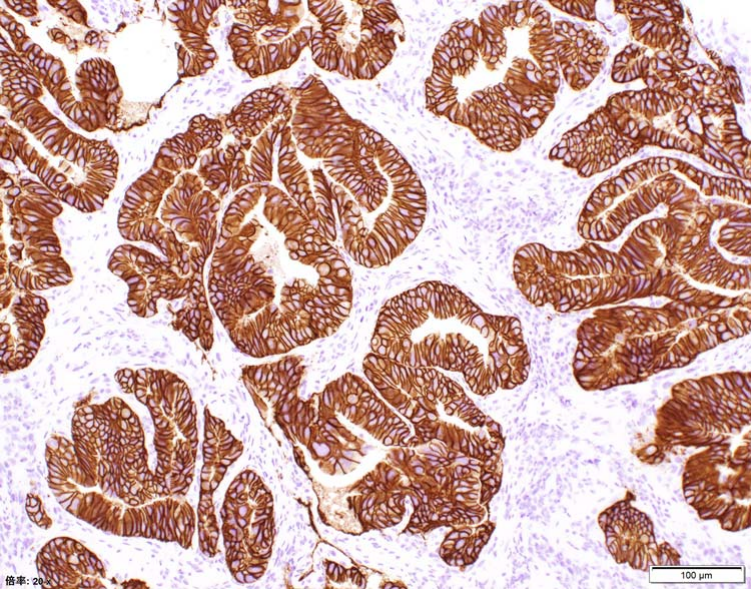
The assessment of the HER2 expression showed that all of the tumor cells had the overexpression of HER2 (Score 3).

Chest and abdominal computed tomography (CT) showed no distant metastasis, but the tumor of the antrum was clearly visualized and was located close to the pancreatic head (Fig. 1). EGD revealed a large ulcerated lesion at the greater curvature of the antrum (Fig. 2). Hematoxylin and eosin staining of the biopsy specimen of the tumor confirmed a well-differentiated adenocarcinoma (tub1, tub2) (Fig. 3). The assessment of the HER2 expression by immunohistochemistry revealed the overexpression of HER2 (3+) in the biopsy tissue of the gastric tumor (Fig. 4).

**Figure 5:**
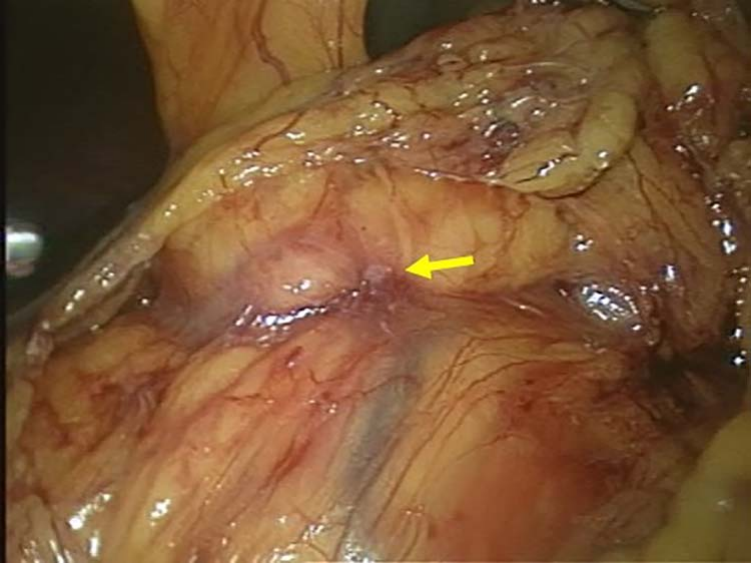
Laparoscopic finding showed that there was no peritoneal dissemination or liver metastasis, but the direct invasion of the gastric tumor to the pancreatic head was suspected (arrow).

Staging laparoscopy showed no peritoneal dissemination or liver metastasis, but direct invasion of the gastric tumor to the pancreatic head was revealed (Fig. 5). The clinical diagnosis was cT4N(+)M0 StageIVA according to the TNM Classification of Malignant Tumors, 8th Edition. We decided to start chemotherapy to shrink the tumor and cause it to separate from the pancreatic head.

**Figure 6:**
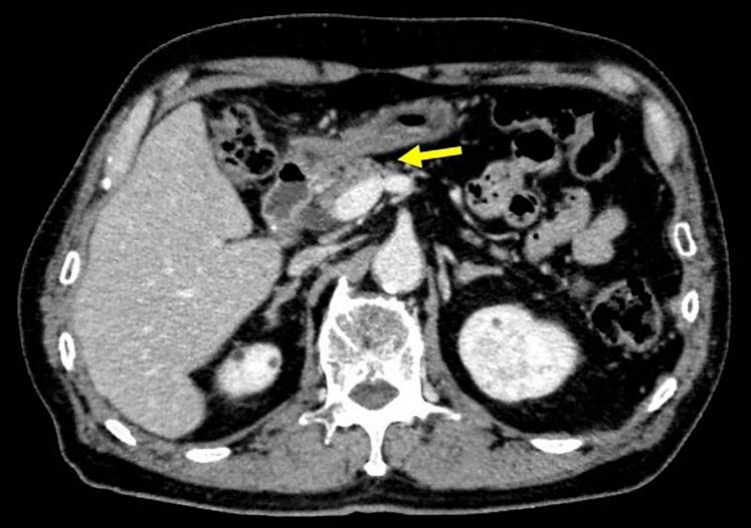
CT after the NAC had been completed showed a significantly shrunken tumor at the antrum that had become detached from the pancreatic head (arrow).

**Figure 7:**
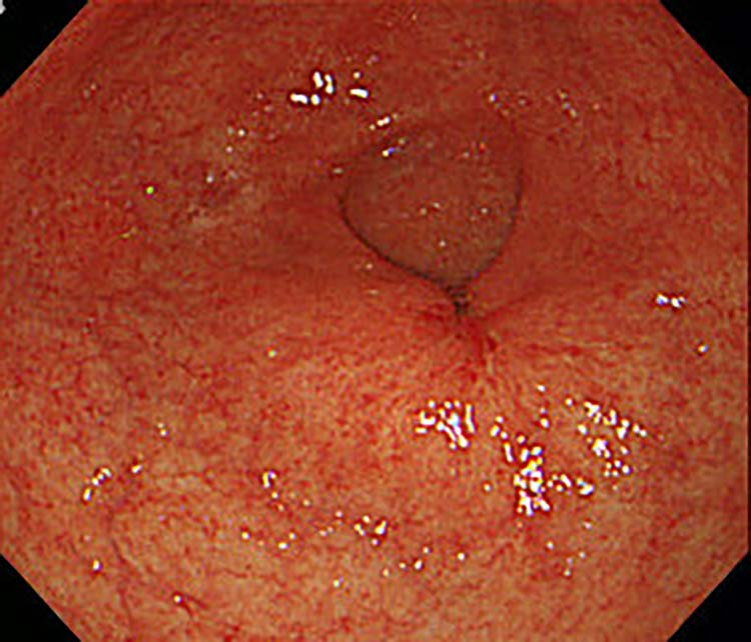
After the four cycles of NAC had been finished, EGD showed a markedly shrunken tumor at the antrum.

The patient received S-1, oxaliplatin and trastuzumab (SOX+Tmab) (S-1; 120 mg/body/day, days 1–14, oxaliplatin; 130 mg/m2, day 1, trastuzumab; 8 mg/kg for the first week and then 6 mg/kg every week thereafter, day 1). After finishing a total of four cycles of neoadjuvant chemotherapy, the efficacy was evaluated by CT, which showed significant shrinkage of the tumor at the antrum and detachment from the pancreatic head, with no enlarged lymph nodes (Fig. 6). Follow-up EGD revealed a very small ulcerative lesion at the antrum (Fig. 7). The serum level of CEA had decreased to 9.0 ng/ml.

**Figure 8:**
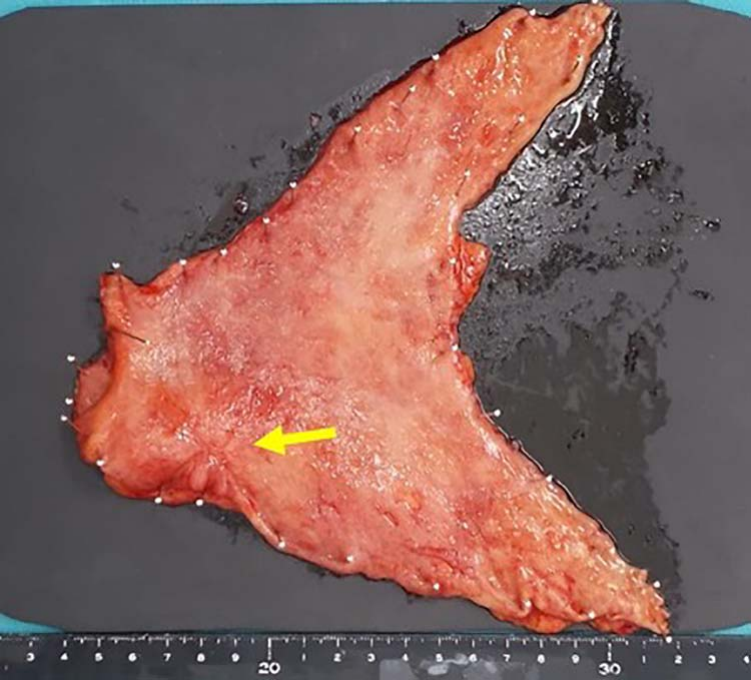
The macroscopic evaluation of the surgical specimen revealed a smaller ulcerative lesion than had been seen preoperatively, with no invasion to the serosa (arrow).

**Figure 9:**
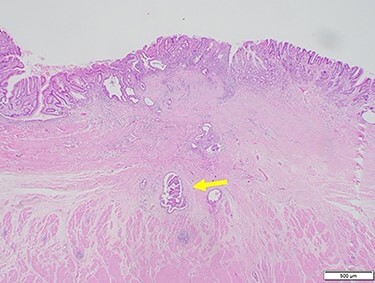
Microscopic findings of hematoxylin and eosin staining of the tumor showed a very small cluster of tumor cells; there was no lymph node metastasis (arrow).

Subsequently, distal gastrectomy with D2 lymph node dissection and Bilroth I reconstruction were performed by laparotomy. Intra-operative exploration showed that the gastric tumor had separated from the pancreatic head, with no obvious metastatic tumors of the liver or peritoneum. The macroscopic evaluation of the surgical specimen revealed a 15 × 13-mm ulcer scar-like lesion at the antrum, not invading the serosa (Fig. 8). The post-operative course was uneventful. The pathological findings were a small 4 × 2 × 2-mm cluster retained in the fibrosis of the submucosal layer and no remnant malignant cells detected at the regional lymph nodes (Fig. 9).

## DISCUSSION

In gastric cancer, achieving complete resection is the goal of treatment, even in cases of locally advanced tumors; however, additional adjacent organ resection with gastrectomy is associated with an increased risk of developing complications [1, 3, 4]. Pre-operative chemotherapy is a promising strategy for improving the resectability of the gastric tumor, removing the need for additional organ resection and suppressing post-operative recurrence [5–7]. We, therefore, attempted pre-operative chemothrapy with the goal of tumor downstaging to prevent the need for PD.

In the present case, the overexpression of HER2 was detected in the gastric tumor. We selected the regimen of SOX+Tmab. The ToGA study was the first international randomized phase III trial of Tmab for gastric cancer comparing cisplatin plus capecitabine or fluorouracil to Tmab plus chemotherapy [8]. That study showed a survival benefit with the addition of Tmab to chemotherapy. The studies of HERBIS-1 and WJOG7212G demonstrated the efficacy of Tmab with S-1 plus cisplatin for HER2-positive gastric cancer [9, 10]. However, regimens containing cisplatin frequently lead to grade 3 or 4 adverse events and require massive hydration to avoid renal toxicity [9, 11].

The G-SOX study was a phase III study evaluating the efficacy and safety of S-1 plus oxaliplatin (SOX) compared with S-1 plus cisplatin (CS) in advanced gastric cancer [12]. SOX showed non-inferiority to CS in terms of the progression-free survival, and it was concluded that SOX was as effective as CS and provided considerable advantages in safety over CS. Furthermore, two multicenter phase II studies demonstrated the efficacy and safety of SOX+Tmab for HER2-positive advanced gastric cancer, so SOX+Tmab is considered a conditionally recommended regimen at the first line for HER2-positive advanced gastric cancer [13, 14].

There have been no randomized control trials (RCTs) or evidence-based regimens of pre-operative chemotherapy for locally advanced gastric cancer. Reddavid *et al.* [15] reviewed the literature for RCTs comparing chemotherapy followed by surgery with surgery alone for gastric cancer. They concluded that none of the trials had a sufficient quality, so larger scale multicenter RCTs comparing the newer regimens, including molecular therapies followed by adequate extended surgery, with surgery alone are needed. We used the SOX+Tmab regimen for the present case of locally advanced gastric cancer because SOX is as effective as CS for gastric cancer, as verified in the G-SOX study, and its tolerability is better than that of CS. In addition, HER2 overexpression was detected in this case, so we used the SOX+Tmab regimen.

In our case, the gastric tumor shrank markedly and separated from the pancreatic head following SOX+Tmab, which enabled preservation of the pancreatic head. This strategy of performing pre-operative therapy lead to safe gastrectomy without post-operative complications. SOX+Tmab is expected to be recognized in the future as a useful pre-operative regimen for locally advanced gastric cancer with the overexpression of HER2. However, no RCTs of NAC for locally advanced gastric cancer have been conducted, and only a few case reports have been published. The accumulation of trials for NAC for gastric cancer is expected.

## AUTHORS’ CONTRIBUTION

M. Sato drafted the manuscript. K.E. and A.H. supervised the writing of the manuscript. M. Sato, K.E., A.H. and M. Shijo provided managements of the patient. All authors read and approved the final manuscript.

## CONFLICT OF INTEREST STATEMENT

None declared.

## FUNDING

The authors received no funding for this study

## CONSENT FOR PUBLICATION

Written informed consent was obtained from the patient for the publication of the case and all accompanying images.
